# A Fast and Cost-Effective Method for Identifying a Polymorphism of Interleukin 28B Related to Hepatitis C

**DOI:** 10.1371/journal.pone.0078142

**Published:** 2013-10-22

**Authors:** Camila da Silva Ferreira, Rodrigo Martins Abreu, Marlone Cunha da Silva, Aline Siqueira Ferreira, Paulo Dominguez Nasser, Flair José Carrilho, Suzane Kioko Ono

**Affiliations:** 1 University of São Paulo School of Medicine, Department of Gastroenterology, São Paulo, SP, Brazil; 2 Federal University of Juiz de Fora, Department of Parasitology, Microbiology and Immunology, Institute of Biological Sciences, Juiz de Fora, Minas Gerais, Brazil; Saint Louis University, United States of America

## Abstract

Approximately 170 million people are chronic carriers of hepatitis C virus (HCV). Patients with chronic hepatitis C are currently treated with pegylated interferon and ribavirin (PEG-IFN/RBV). A genome-wide association with PEG-IFN/RBV treatment response and a single nucleotide polymorphism (rs12979860) has been identified near the interleukin 28B gene that encodes interferon-λ-3. In this paper, we describe an innovative, fast, and low-cost multiplex polymerase chain reaction with confronting two-pair primers that detects the rs12979860 polymorphism. The assay is internally controlled and does not require the use of restriction endonucleases or special equipment. Moreover, the assay decreases costs, being about 40% cheaper than direct sequencing methods.

## Introduction

 Approximately 170 million people are chronic carriers of hepatitis C (HCV), which is the leading cause of liver transplants worldwide [1]. The standard treatment for patients with chronic infection is the combination of pegylated interferon α 2a/2b (PEG-INF) and ribavirin (RBV) [2]. Several criteria are used for the prognosis of the disease, including genotype, viral load, age, sex, and hepatic fibrosis [3]. 

Explanations for the differences in treatment response or for spontaneous HCV elimination have been widely investigated [4]. Among these efforts, the role of genetic polymorphisms was recently highlighted. A genome-wide association study showed a strong association of the single nucleotide polymorphism (SNP) rs12979860, located near the gene for interleukin 28B (IL28B), and sustained virological response (SVR), which is the absence of detectable virus at the end of a follow-up evaluation [5]. Another study described an association of this SNP with the spontaneous clearance of the virus [6]. 

Currently, the polymorphism is analyzed mostly by custom-designed TaqMan assays [7-9]. These assays as well as direct sequencing and other assays [10-12] allow for the screening of patient samples. However, for many laboratories, large routine analysis in a tertiary care hospital with reagent and equipment costs limits these implementations, especially in developing countries.

Here we introduce a fast and low-cost alternative method of identifying the IL28B polymorphism. Polymerase chain reaction (PCR) with confronting two-pair primers (CTPP) was developed in the year 2000 with the goal of being simpler than PCR–restriction fragment length polymorphism analysis and has been standardized regarding eliminated steps, all of which could save time and costs in rs12979860 genotyping.

## Methods

### Samples and DNA isolation

 Peripheral blood samples were collected from patients with chronic hepatitis C who were being treated or had been previously treated with pegylated interferon α 2a/2b (PEG-INF) and ribavirin (RBV). All patients were under care at the Gastroenterology Department at the Hospital das Clínicas da Faculdade de Medicina da Universidade de São Paulo, Brazil. This study was approved by the Hospital das Clinicas Ethics Committee (São Paulo, Brazil), and all patients signed the informed consent. 

Genomic DNA was extracted from buffy coat using the *QIAamp DNA Blood Mini Kit* (Qiagen, Hilden, Germany) and stored at -20°C [13].

### PCR-CTPP assay

 The PCR CTPP technique was presented by Hamajimae employees in 2000, with a purpose to be simpler than the PCR-RFLP, but keeping the cost low [14]. The principle of PCR-CTPP lies in designing a pair of primers for each allele. The reference sequence for chromosome 19, clone CTC-246B18, deposited in GenBank under code number AC011445, was used for primer design. Primers are shown in Table 1.

**Table 1:** Primers.

**Primers**	**Primer sequence (5'–3')**	**Length (bp)**
IL28B_F1	GAC GAG AGG GCG TTA GAG CG	20
IL28B_R1	GGA GTG CAA TTC AAC CCT GGT TC**G**	24
IL28B_F2	GAG CTC CCC GAA GGC G**T**	17
IL28B_R2	AAC GCA GGC TCA GGG TCA AT	20

Bold: The base 3 'reverse primer (R1) is complementary to the base cytokine (C). the base 3 'primer stream (F2) is complementary to thymine base (T)

**Figure 1:**
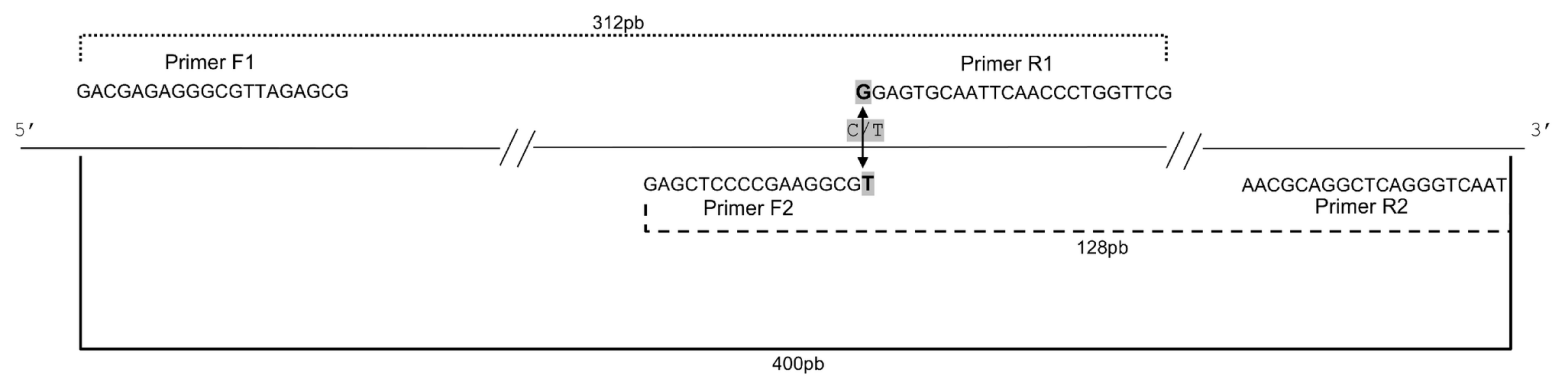
Design of the primers: 312 bp, ······, primer-amplified fragments corresponding to allele C. 128pb, ▬ ▬ , primer-amplified fragments corresponding to the allele T. In ▬ ▪ ▬ , fragment generated by primers F1 and R2 representing the positive control.

With the first primer pair, F1 and R1, the 3’ base of reverse primer (R1) is the base complement to cytosine (C) and therefore amplifies only the C allele, generating a fragment of 312 bp (base pairs). With the second primer pair, F2 and R2, the 3’ base of the forward primer (F2) has a thymine base (T) and therefore amplifies only the T allele. F2 and R2 produce a fragment of 128 bp. A 400-bp fragment is generated by primers F1 and R2. It is a fragment common to all reactions and is considered the reaction positive control (Figure 1).

The reactions consisted of a total volume of 50 µl containing 1.2 U of Taq Gold (Applied Biosystems, USA), 0.5 µM of primers F1, F2, and R2, 0.75 µM of primer R1, 1.0 µM of dNTP, 5.0 mM of MgCl_2_, and 70–100 ng of genomic DNA. Thermocycler conditions were as follows: preheating at 95°C for 5 min followed by 40 cycles of 95°C for 30 s, 69.2°C for 1 min, and 72°C for 3 min.

**Figure 2:**
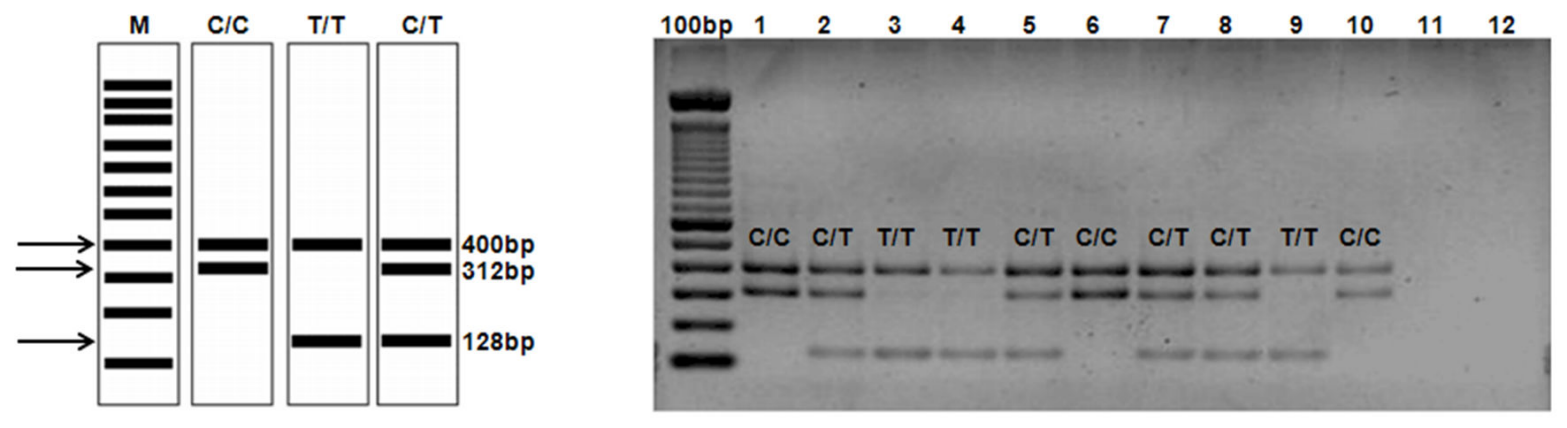
Standardization of PCR-CTPP. A - Graphic of the expected result in gel electrophoresis. B - The genotype analysis was performed by differentiatingthe size of fragments; the homozygous C/C gives bands of 400 bp and 312 bp, the homozygous T/T gives bands of 400 bp and 128 bp, and the heterozygote genotype yields bands of 400 bp, 312 bp, and 128 bp. The lanes 1 to 3 represent positive controls. The shafts 4 to 10 represent samples and 11 and 12 negative controls.

PCR products were separated by standard electrophoresis on 2% agarose gels containing ethidium bromide. The PCR-CTPP was standardized using genomic DNA from 11 samples (Figure 2). All samples were previously genotyped by direct sequencing and real time PCR. Sequencing reactions were performed with the BigDye Terminator Cycle Sequencing kit and submitted on an ABI Prism 3100 Genetic Analyser (Applied Biosystems, CA, USA). The rs12979860 was genotyped using the ABI TaqMan SNP genotyping assays (Applied Biosystems, Foster City, CA, USA) [15]. 

Negative and positive controls were included in all reactions to ensure reproducibility of results.

### Cost analysis

 We also evaluated the cost benefit of PCR-CTPP. We compared genotyping cost and time for the two methods of PCR-CTPP, direct sequencing and real time PCR. Estimation of unitary test cost was based on the currently available commercial reagent prices in Brazil, where the study was conducted. Equipment maintenance, human resources, and other indirect costs were not taken into account for comparison and calculation [16].

## Results and Discussion

 The polymorphism rs12979860 (IL28B) has been studied extensively throughout the world. Several reports have described a strong association between the response to treatment and IL28B genotype [5,6,17,18]. Genotyping of IL28B SNPs provides valuable information about the possibility of the patient’s achieving a sustained virological response to treatment of chronic hepatitis C, which is increasingly important in clinical practice.

Today, the IL28B genotype is included in the American Association for the Study of Liver Diseases and the European Association for the Study of the Liver guidelines [19,20]. As a result, physicians should consider testing IL28B in patients with hepatitis C; however, the implementation of routine genotyping has been halted in tertiary care hospitals because of the need for molecular biology tools that are expensive and highly complex.

The CTPP multiplex assay described here detects in a single reaction the genotypes C/C, C/T, and T/T. All samples evaluated with CTPP (n = 211) presented agreement with sequencing. The analysis of 71 samples with real time PCR also presented agreement with the genotype as demonstrated in table 2. The PCR-CTPP was validated in 211 patients with chronic hepatitis C; Table 3 shows the IL28B genotyping and the results of standard treatment with PEG-IFN/RBV.

**Table 2:** distribution of genotypes according to the methods.

	**Genotype**			
**Method**	**CC**	**CT**	**TT**	**TOTAL**
PCR CTPP	68	106	37	211
Direct sequencing	68	106	37	211
Real-time PCR	26	37	9	72

**Table 3:** Clinical characteristics of 211 patients with chronic hepatitis C, treatment with standard PEG-IFN/RBV, according to the rs12979860 genotype.

**rs12979860 genotype**				
	**CC (%)**	**CT (%)**	**TT (%)**	**p**
**Age**	51.1 ± 10.6	50.9 ± 11.8	56.2 ± 9.4	0.024
**BMI**	27.0 ± 4.4	26.7 ± 5.0	27.2 ± 4.4	ns
**SVR**	29 (52.7)	21 (38.2)	5 (9.1)	<0.001
**Non SVR**	39 (25.0)	85 (54.5)	32 (20.5)	< 0.001

SVR: sustained virological responseBMI: body mass index

This PCR-CTPP reaction showed little variation when performed by different technicians, confirming the validity of the methodology. The inter-technician kappa’s ranged from 0.92 to 1.0, indicating excellent inter-technician reliability (Kappa’s obtained from 22 samples). We used three positive controls (a sample of each genotype) to ensure the reliability of results. 

The cost of each reaction was US$ 184,12, US$ 174,56 and US$ 31.57 for the direct sequencing, Real time PCR and PCR-CTPP respectively. 

Regarding the time, sequencing took an average of 850 minutes, the real time PCR took an average of 310 minutes and the PCR-CTPP an average of 460 minutes.

Only the direct cost of reagents was analyzed. However, if we had considered the cost of equipment, maintenance and labor the difference between the costs of methodologies would be even less for PCR CTPP, as it uses only a thermocycler and a set of electrophoresis .

## Conclusion

 In conclusion, this method allows rapid genotyping of the polymorphism rs12979860, which is reproducible in minimally equipped laboratories; it does not require any special equipment and is a relatively low-cost procedure. The PCR-CTPP method can be used for large testing arrays and is also suitable for genotyping a small number of samples.

## Supporting Information

Figure S1 **Genotype C/C, shown in blue color is closer to the y axis, the T/T genotype represented by red color is closest to x-axis and genotype C/T, represented by the color green, lies between the two shafts.** The negative controls and samples that did not amplify any are close to the point 0 the graph, as shown.
(TIF)
