# Supplementary figures and images for: A Fast and Cost-Effective Method for Identifying a Polymorphism of Interleukin 28B Related to Hepatitis C
Source: PLoS One. 2013 Oct 22;8(10):e78142. doi: 10.1371/journal.pone.0078142 (PMC3805516; doi:10.1371/journal.pone.0078142)

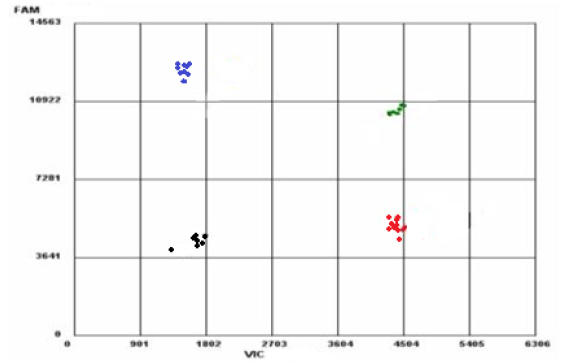

Supplement: Figure S1 — Genotype C/C, shown in blue color is closer to the y axis, the T/T genotype represented by red color is closest to x-axis and genotype C/T, represented by the color green, lies between the two shafts. The negative controls and samples that did not amplify any are close to the point 0 the graph, as shown. (TIF) [file pone.0078142.s001.tif]
